# Antimicrobial Nanofiber Based Filters for High Filtration Efficiency Respirators

**DOI:** 10.3390/nano11040900

**Published:** 2021-04-01

**Authors:** Maria Pardo-Figuerez, Alberto Chiva-Flor, Kelly Figueroa-Lopez, Cristina Prieto, Jose M. Lagaron

**Affiliations:** 1 Novel Materials and Nanotechnology Group, Institute of Agrochemistry and Food Technology (IATA), Spanish Council for Scientific Research (CSIC), Calle Catedrático Agustín Escardino Benlloch 7, 46980 Paterna, Spain; mpardo@iata.csic.es (M.P.-F.); kjfigueroal@iata.csic.es (K.F.-L.); cprieto@iata.csic.es (C.P.); 2 Bioinicia S.L., R & D Department, Calle Algepser, 65 Nave 3, 46980 Paterna, Spain; achiva@bioinicia.com

**Keywords:** nanofibers, respirators, antimicrobials, SARS-CoV-2, electrospinning

## Abstract

Electrospinning has been used to develop and upscale polyacrylonitrile (PAN) nanofibers as effective aerosol filtration materials for their potential use in respirators. The fibers were deposited onto non-woven spunbond polypropylene (SPP) and the basis weight (grammage, g/m^2^) was varied to assess the resulting effect on filtration efficiency and breathing resistance of the materials. The results indicated that a basis weight in excess of 0.4 g/m^2^ of PAN electrospun fibers yielded a filtration efficiency over 97%, with breathing resistance values that increased proportionally with the amount of basis weight added. With the aim of retaining filter efficiency whilst lowering breathing resistance, the basis weight of 0.4 g/m^2^ and 0.8 g/m^2^ of PAN electrospun fibers were strategically split up and stacked with SPP in different configurations. The results suggested that a symmetric structure based on SPP/PAN/PAN/SPP was the optimal structure, as it reduces SPP consumption while maintaining an FFP2-type of filtration efficiency, while reducing breathing resistance, specially at high air flow rates, such as those mimicking FFP2 exhalation conditions. The incorporation of zinc oxide (ZnO) nanoparticles within the electrospun nanofibers in the form of nanocomposites, retained the high filtration characteristics of the unfilled filter, while exhibiting a strong bactericidal capacity, even after short contact times. This study demonstrates the potential of using the symmetric splitting of the PAN nanofibers layer as a somewhat more efficient configuration in the design of filters for respirators.

## 1. Introduction

The unexpected appearance and rapid spreading of the severe acute respiratory syndrome coronavirus (SARS-CoV-2) at the end of 2019, has affected over 14 million people and caused over a million deaths all over the world as of November 2020 [1,2,3]. The SARS-CoV-2 belongs to the coronavirus family, a type of positive sense single-stranded RNA virus with a spike of glycoproteins on its envelope which gives the virus a crown-like morphology, with a size ranging from 60 to 140 nm [4,5]. The first reported cases indicated that SARS-CoV-2 affects mainly the respiratory tracks, with patients reporting mostly fever, cough, loss of smell and taste, headache and fatigue [6]. The exported cases from the epicenter of the pandemic also suggested that human-to-human transmission occurs rapidly. It is believed that virus spreading occurs through different means, being the inhalation of mouth-expelled microdroplets of over 5 μm and aerosols below 5 μm, as the most recently recognized mechanism [1,5]. Due to the fast spreading, authorities all over the globe have focused on reducing the transmission by practicing isolation, social distancing and the use of face masks and respirators [5,7]. Although isolation measures have proven effective, countries cannot be confined for a long term due to socio-economic reasons [8] and therefore, authorities have recommended, and even declared compulsory [9], the use of face masks and respirators to avoid the spreading of such global infections whilst avoiding recession and financial collapse.

In light of this, there is an urgent need to generate highly efficient filtration materials that can help from a prophylactic view-point to fight the current pandemic [1,5]. Current commercial filters are mainly microfiber-based materials, being the most efficient meltblown polypropylene. Although frequently used, meltblown polypropylene presents uncertain durability while in use, due to moisture-induced dissipation of the electrostatic filtration mechanism in which the technology is based [10,11,12].

Electrospinning is becoming an emerging technology in the area of filtration to generate nanofiber-based filter materials. These fibrous materials at the nano scale have significant morphological advantages over current conventional filters, such as fiber diameters within the nanoscale, homogeneous and interconnected small pore size, good mechanical properties and a high specific surface area of the fibers. These characteristics favor a mechanical filtration mechanism, where interception and Brownian diffusion retention of small particles between 50–5000 nm result in highly efficient filtering materials [11,13]. Due to these features, the nanofiber technology also provides a high filter efficiency at very low grammages, structural simplicity and the possibility of functionalization of the nanofibers to add new active performances. This makes this technology cost-effective, competitive and more functional when compared to, for instance, electrostatic meltblown microfibers used in conventional filters [11].

Electrospun nanofibers are normally deposited onto a substrate, commonly a non-woven fabric [14]. Once an optimum substrate has been chosen, electrospun fibers can be designed to increase filter efficiency whilst maintaining a low breathing resistance. This can be typically achieved either by modification of interfiber porosity or by increasing the basis weight of the nanofibers layer. The latter may not be an optimal strategy since a larger nanofiber mass deposition will lead to a higher filter efficiency but also to a higher pressure drop, obtaining poor breathability. Various strategies have been presented in the literature to try and achieve efficient filtration nanofiber materials. In a study from Leung and Sun [15], the filtration efficiency of untreated and corona-treated (charged) polypropylene deposited-polyvinylidene fluoride (PVDF) fibers with a size diameter of 450 nm was analyzed. The study confirmed that corona treatment could favor high filtration efficiency due to high charge densities on filters, leading to a stronger electrostatic mechanism. However, filter performance did not increase when the authors attempted to increase the filter basis weight, and so to enhance the filter electrical mechanisms, the filter was divided into multiple layers. The stacking up of the filter not only increased filter efficiency, but also lowered pressure drop. This effect was more notable at higher basis weight, leading to an overall increase on the quality factor of the filter [15]. 

Similarly, filters of PVDF electrospun fibers were produced with different fiber diameters (84, 191, 349 and 525 nm) and were also corona-discharged to enhance differences between mechanical and electrostatic capture [16]. Filter efficiency and pressure drop were assessed with sodium aerosols against single and multi-nanofiber layers deposited in polypropylene, by stacking each nanofiber with its SPP substrate in several configurations, ranging from 2 to 8 layers. The authors observed that using the multilayer approach reduced pressure drop significantly when compared to the monolayer structures at the same basis weight. It was hypothesized that this could be attributed to the introduction of macropores from the substrate material layer to the filter, which would interrupt the micropore structure of the multilayer nanofibers and thus reduce the fiber packing density. The benefits of the multilayer structure versus the monolayer could be demonstrated for the four different fiber diameters tested, although changes in the basis weight of the modules had to be adjusted for each fiber diameter [16].

Among the different types of polymers used in filtration, polyacrylonitrile (PAN) has become a good candidate due to its good mechanical properties and hydrophobicity, as well as both its thermal and chemical stability [17,18,19]. A comparison of the filtration performance between single and bilayer electrospun filters on a polyester–viscose non-woven substrate was also analyzed for the PAN fibers. In this case, the bilayer structure consisted of a layer of smooth fibers with 400 nm diameter, and another layer containing beaded structures (beads, 200 nm, strings 55 nm). The change in morphology across the layers altered the thickness and the packing density of the filter. Interestingly, the order of the beaded bilayer also influenced filter performance. When the beaded fiber layer was on the upper layer of the filter, filtration efficiency was above 95% and the pressure drop was 137 Pa. When the beaded fiber layer was placed in the inner layer, penetration performance was similar (95%); however, the pressure drop reached 112 Pa [11]. The same group carried out a similar study in which the bilayer structure was made of smooth nanofiber layers of 200 and 400 nm fiber diameters over a polyester-viscose substrate. The bilayer structures proved beneficial over single layer structures and it was observed that the stacking order in the bilayer could influence the filter performance. The bilayer structure with 400 nm fiber diameter at the top and the 200 nm fiber diameter layer at the bottom of the filter showed a significant reduction in pressure drop (from 208 Pa to 87 Pa) but comparable filtration efficiency [20]. 

PAN electrospun fibers have been used on its own as filtration membranes, but this material has also been conveniently additivated with certain agents to enhance fiber functionality, especially in the field of sensors, filtration, catalysis, or food packaging [21,22,23]. Components such as silver (Ag) [24,25], titanium dioxide (TiO_2_) [26], palladium (Pd) [27], zinc oxide (ZnO) [28,29], or copper (Cu) [30] have shown good antimicrobial properties against a large variety of microorganisms such as bacteria, fungi, and viruses [31,32] and can be incorporated in the electrospun material in the form of nanocomposites. In a recent study, Bechelany et al. [23] prepared TiO_2_/PAN, ZnO/PAN, and Ag/PAN nanofibers over polyethylene terephthalate (PET) substrates for their use as air filters. The presence of nanoparticles did not alter the filtration efficiency, as all of them had a filtration above 95%. Additionally, the Ag/PAN filter was the most optimum filter as it showed a high filtration efficiency (>98%) and a low pressure drop (68.13 Pa), as well as very good antibacterial properties. The combination of electrospun fibers with antimicrobial components present a promising prospect in obtaining a highly efficient filtration materials that are able to prevent microorganism growth and contamination, thus protecting the citizens from microbial pollution [30]. 

This paper reports work carried out to develop high throughput antimicrobial electrospun filter structures made of PAN nanofibers. To do this, we characterized first the filtration efficiency and breathing resistance as a function of the increasing PAN nanofibers bases weight. Furthermore, we proposed the use of the innovative approach of symmetrical splitting up of the PAN electrospun nanofibers to yield an optimum balance between the pressure drop (breathability) and the filtration of the material. To impart biocide performance, the PAN electrospun fibers were loaded with ZnO nanoparticles, exhibiting antimicrobial activity without compromising filtration efficiency. The herein developed filtration membranes could open up new avenues for the development of the next generation of ultrahigh filtration materials. Finally, the development and manufacturing of these membranes were obtained in a high throughput industrial equipment, which further indicates that mass production of such materials is feasible.

## 2. Materials and Methods

### 2.1. Materials

Polyacrylonitrile (PAN, Mw 150.000, 99%) was purchased from Carbosynth (Compton, United Kingdom). Zinc Oxide (CR-4FCC1), 99% purity, specific surface of 4.5 m^2^/g, bulk density of 40 lb/ft^3^, and specific gravity of 5.6 were obtained from GH Chemicals LTD^®^ (Quebec, Canada) and N, N-Dimethylformamide (DMF, 99%) was purchased from VWR Chemicals (Leuven, Belgium). All the electrospun fibers were deposited over a porous non-woven spunbond polypropylene material of 30 g/m^2^ (SPP, NVEVOLUTIA, Valencia, Spain). All reagents were used as received without further purification.

### 2.2. Solution Preparation and Characterization 

Polyacrylonitrile was mixed with DMF at a concentration of 11 wt.% and dissolved by stirring at 450 rpm and at 50 °C overnight. For the nanocomposites fibers, solutions of PAN with DMF (11 wt.%) containing 1 wt.% (PAN-ZnO1), 3 wt.% (PAN-ZnO_3_), 10 wt.% (PAN-ZnO10) and 20 wt.% (PAN-ZnO20) of ZnO were stirred overnight and homogenized in a high power ultrasound SONOPULS HD 2200.2. (Scharlab, Barcelona, Spain) at 40% power for 2 min.

The apparent viscosity (ηa) was determined using a rotational viscosity meter Visco BasicPlus L from Fungilab S.A. at 50 rpm with a L3 spindle (Barcelona, Spain). The conductivity was evaluated using a conductivity meter Seven2Go™ from Metler Toledo 742-ISM (Barcelona, Spain). All the measurements were carried out in triplicate at room temperature.

### 2.3. Electrospinning

The electrospun PAN fibers mat and its antimicrobial nanocomposites were processed using a Fluidnatek LE-500 production equipment with an industrial 50 cm wide roll-to-roll system. The solutions were pumped through a linear multinozzle injector at a feed rate of 1 mL/h per emitter, applying a voltage of 30 kV at the emitter and a voltage of −10 kV at the collector. The fibers were collected onto the spunbond polypropylene (SPP) at a distance of 20 cm under controlled environmental conditions of 30 °C and 30% RH (relative humidity). The conditions for electrospinning PAN-ZnO were the same as mentioned above for the pristine PAN electrospun mats. To obtain the different basis weight of the mats (grammage, g/m^2^), the deposition time was optimized and adjusted accordingly. 

### 2.4. Mat Morphology 

The morphology of the electrospun fibers was determined by a field emission scanning electron microscope (FE-SEM) using a Hitachi TM-4000 (Tokyo, Japan) with an electron beam acceleration of 10 kV. Analysis of the fiber diameter was carried out with the ImageJ software (version 1.52, National Institutes of Health, Bethesda, MD, USA). At least 100 fibers were randomly selected and measured from three different images. The average fiber diameter as well as the standard deviation (SD) were calculated and are presented in the corresponding Figures. 

The dispersion of ZnO in the matrix of the fibers was analyzed by transmission electron microscopy (TEM) using a JEOL JEM 1010 (JEOL Ltd., Tokyo, Japan) with an acceleration voltage of 100 kV. The fibers were collected on a sandwich-type holder (Agar Scientific-G230, Agar Scientific Ltd., Stansted, UK) with a mesh size of 3.05 mm.

Porosity of the fiber mats was determined by cutting out sample sizes of 3 × 3 cm^2^, obtaining the mass with a Mettler Toledo MS105 balance and the thickness by measuring the cross-section of the samples with a field emission scanning electron microscope (FE-SEM) using a Hitachi TM-4000 (Tokyo, Japan). The apparent density was then measured with the collected data (see Equation (1)) and the porosity was determined using the bulk density of PAN (1.184 g/cm^3^, see Equation (2)):

(1) $$Apparent\;density=\frac{weight\;of\;the\;mat}{thickness*area\;of\;the\;mat}$$

(2) $$Porosity\;\left(\%\right)=\left(1-\frac{Apparent\;density}{Bulk\;density}\right)\times 100$$

### 2.5. Antimicrobial Activity 

Antimicrobial performance was analyzed for the composite electrospun filters. The strains *Staphylococcus aureus* CECT240 (ATCC 6538p) and *Escherichia coli* CECT434 (ATCC 25922) were obtained from the Spanish Type Culture Collection (CECT, Valencia, Spain). The bacterial strains were stored in phosphate-buffered saline (PBS) with 10 wt.% tryptic soy broth (TSB) obtained from Conda Laboratories (Madrid, Spain) and 10 wt.% glycerol at −80 °C. Previous to each study, a loopful of bacteria was transferred to 10 mL of TSB and incubated at 37 °C for 24 h. A 100 µL aliquot from the culture was again transferred to TSB and grown at 37 °C to the mid-exponential phase of growth. The optical density showing an absorbance value of 0.20 and measured at 600 nm in a UV–Vis spectrophotometer (VIS3000 Dinko Instruments, Barcelona, Spain) determined that the initial bacterial concentration was approximately a 5 × 10^5^ colony-forming unit (CFU)/mL. The antimicrobial performance of PAN filters containing ZnO particles (1, 3, 10 and 20 wt.%) was determined based on the guidelines of the Japanese Industrial Standard JIS Z2801 (ISO 22196:2007) [33]. A microorganism suspension in TSB of *S. aureus* and *E. coli* was applied to a 5 cm × 5 cm material sample containing PAN only (negative control), and PAN-loaded ZnO particles at 1, 3, 10, and 20 wt.% (test material). Thereafter, the inoculated samples were placed in open bottles and incubated for 24 h at 24 °C and at a relative humidity (R.H.) of at least 95%. For the PAN containing a 3 wt.% of ZnO, samples were also incubated for 1, 3, 6, and 8 h. Bacteria were recovered with PBS, 10-fold serially diluted and incubated for 24 h at 37 °C to quantify the number of viable bacteria by a conventional plate count. The antimicrobial activity reduction (R) was evaluated at 24 h using Equation (3):

(3) $$R=\left[Log\left(\frac{B}{A}\right)-Log\left(\frac{C}{A}\right)\right]=Log\left(\frac{B}{C}\right)$$

where *A* is the average of the number of viable bacteria on the control sample immediately after inoculation, *B* is the average of the number of viable bacteria on the control sample after 24 h, and *C* is the average of the number of viable bacteria on the test sample after 24 h. The antibacterial activity of the filters was assessed as a non-significant reduction if R < 0.5, a slight reduction if R ≥ 0.5 and <1, a significant reduction when R ≥ 1 and <3, and a strong reduction if R ≥ 3. Experiments were performed in triplicate [34].

### 2.6. Filter Performance

The filtration and breathing resistance performance of the filter was measured using the procedures gathered within the European EN149:2001+A1:2009 standard using a Filter Media Test Rig PMFT 1000 M (Palas^®^, Karlsruhe, Germany). The penetration test was carried out with a paraffin oil aerosol with particle sizes spanning from ca. 0.140 to 4 µm, after 3 min of aerosol exposure. The pressure drop of the samples, named in this work as breathing resistance (Pa), was measured by passing compressed air through the filter at flow rates of 30 and 95 L/min simulating inhalation, and at 160 L/min simulating exhalation in the opposite direction. The dimension of the samples measured was approximately 50 cm^2^ and the testing conditions were 24 ± 2 °C and 50 ± 10% relative humidity. 

According to the abovementioned EN149 standard, final respirators should have a filter efficiency of 94% or higher to the paraffin aerosol and a pressure drop limit of 70 Pa at 30 L/min, of 240 Pa at 95 L/min during inhalation, and of 300 Pa at 160 L/min during exhalation, to accredit FFP2 like performance. FFP2 or its equivalent is the most recommendable filter according to the Health Authorities to protect, especially for healthcare professionals, from COVID-19 [35]. Since pressure drop has a linear relationship with the sample area and most commercial respirators have respiration areas bigger than 200 cm^2^, the breathing resistance data measured in the 50 cm^2^ sample areas should be effectively divided by a factor of at least 4 to assess their compliance with FFP2 performance. 

## 3. Results and Discussion

### 3.1. Fiber Morphology

**Figure 1:**
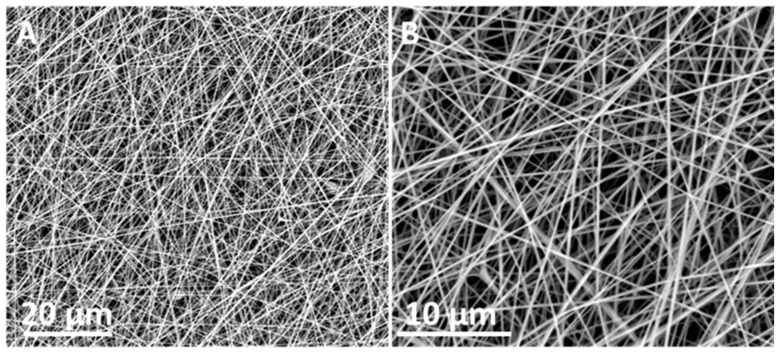
(**A**) Field emission scanning electron microscope (FE-SEM) micrographs of polyacrylonitrile (PAN) electrospun fibers at 3000×. (**B**) Field emission scanning electron microscope (FE-SEM) micrographs of polyacrylonitrile (PAN) electrospun fibers at electrospun fibers at 8000×.

Figure 1 shows the morphology of the generated PAN fibers by FE-SEM. Initially, electrospun mats were deposited onto non-porous substrates until electrospinning parameters were optimized. The images show randomly oriented, ultrafine and smooth fiber morphologies with an average diameter within the range of 200–300 nm.

**Figure 2:**
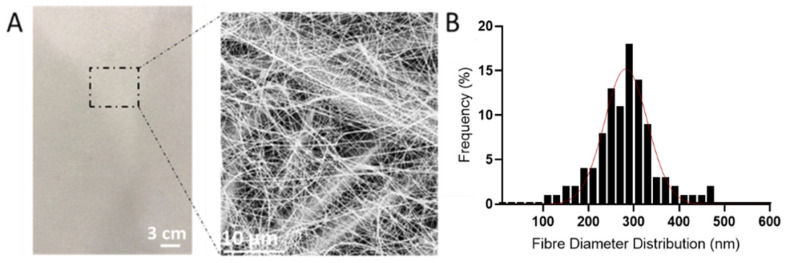
(**A**) Macroscopic and microscopic images of the spunbound polypropylene (SPP) coated with the with polyacrylonitrile (PAN) electrospun fibers. (**B**) Average fiber distribution of polyacrylonitrile (PAN) electrospun fibers.

The optimized electrospinning parameters were utilized to deposit PAN nanofibers onto the non-woven polypropylene substrate (SPP). Figure 2A shows the morphology, both at the macroscopical and microscopical level, of the electrospun fibers deposited onto the SPP. Smooth nanometric fibers were obtained on top of the SPP microfibers, which can be discerned due to the low basis weight deposition of the nanofibers (0.4 g/m^2^). Figure 2B represents the fiber size distribution of the electrospun fibers onto the non-woven substrate, indicating an average size of 282.5 ± 47.6 nm. The use of nanometric fibers have proven advantageous in air filtration, since their small diameter and high surface to volume ratios, can enhance the capture of particles through interception [13,16]. As well as this, the homogeneous porosity and slip flow effect of the nanofibers will certainly result in a lower pressure drop than their microfibers counterparts [36]. These characteristics make electrospun nanofibers very attractive in the filtration field. 

### 3.2. Filter Performance Using Mono and Multilayer Electrospun Fibers

**Figure 3:**
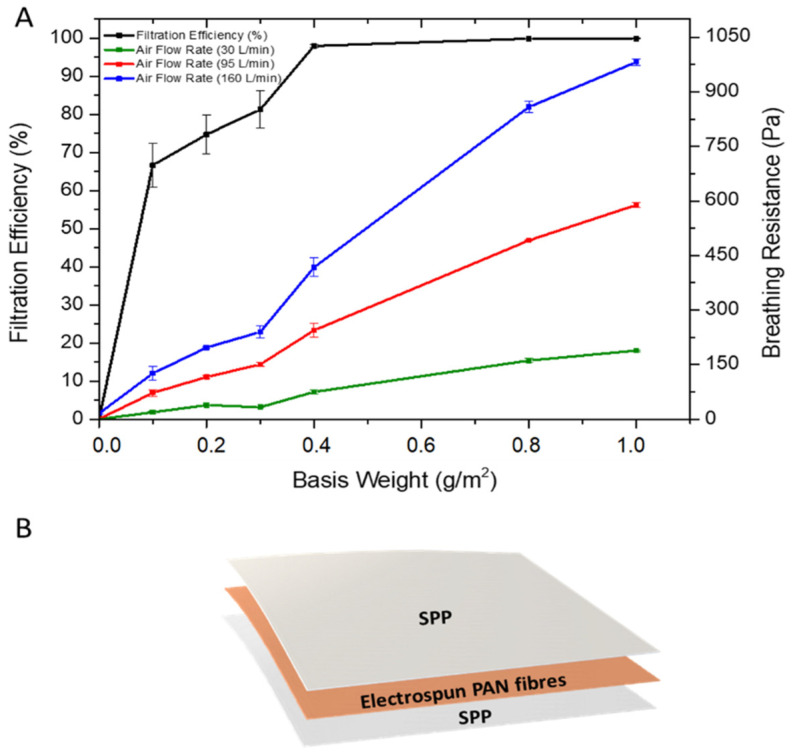
(**A**) Filtration efficiency and breathing resistance of control (SPP/SPP) and monolayer spunbond polypropylene/polyacrylonitrile/spunbond polypropylene (SPP/PAN/SPP) filter with various nanofiber basis weights (g/m^2^). Results are presented as the mean ± SD (*n* = 3). (**B**) Diagram of spunbond polypropylene/polyacrylonitrile/spunbond polypropylene (SPP/PAN/SPP) filters with electrospun nanofibers.

It is known that when the basis weight is increased in filters, dramatic changes in filter efficiency may occur, especially for nanofibrous filters, where a low basis weight is required for high filtration performance and low pressure drop [16,20,37,38]. In fact, the use of nanofibers in air filtration applications requires much less basis weight than the microfibrous-based filters [11,16,39] and hence, the optimum basis weight of PAN fibers to obtain a high filter efficiency and low breathing resistance was investigated (Figure 3A). To do this, a mass deposition of nanofibers ranging from 0.1–1 g/m^2^ were deposited on an SPP by electrospinning. Thereafter, another SPP was placed over the electrospun deposition to protect the PAN fibers and to provide better structural rigidity to the filter (Figure 3B) [17]. Figure 3A shows the relationship between the basis weight against the filter efficiency and breathing resistance. Initially, two layers of SPP/SPP without nanofibers were measured as the reference system. The filter efficiency was very poor, with a value of approximately 2.62 ± 1.78%. In terms of the breathing resistance, the value was below 20 Pa for all the flow rates tested. Once the PAN electrospun fibers were added to the substrate, an increase in filtration efficiency of up to 60–70% was observed by using only 0.1 g/m^2^. As the basis weight increased, so did the filter efficiency, reaching a 97% at 0.4 g/m^2^ and maintaining between 97–99% for the 0.8 and 1 g/m^2^ basis weight. As expected, the higher the filtration efficiency, the higher the breathing resistance achieved for the three different air flows (30, 95 and 160 L/min), with values reaching their maximum at 1 g/m^2^ basis weight. It should be borne in mind that testing was done in filter areas of 50 cm^2^; thus, although filtration efficiency should not, and has not been seen (i.e., 50 vs. 100 cm^2^, results not shown), to depend on filter area, breathing resistance is expected to decrease linearly with the increasing sample area. Respirators for personal protection typically have areas larger than 200 cm^2^, so to anticipate the actual breathing resistance on a typical final respirator, these values should be divided by, at least, a factor of 4. 

**Figure 4:**
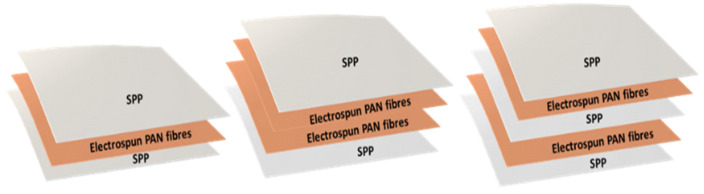
Schematic of the mono and multilayer structures of spunbond polypropylene/polyacrylonitrile/spunbond polypropylene (SPP/PAN/SPP), spunbond polypropylene/polyacrylonitrile/polyacrylonitrile/spunbond polypropylene (SPP/PAN/PAN/SPP) and spunbond polypropylene/polyacrylonitrile/spunbond polypropylene/polyacrylonitrile/spunbond polypropylene (SPP/PAN/SPP/PAN/SPP) filters, prepared with PAN electrospun nanofibers.

Filter efficiency and, therefore, breathing resistance, can be tailored by either modifying the fiber diameter, packing density and/or the fiber morphology of the electrospun mat. In this case, increasing the deposited mass of nanofibers with the same fiber diameter increased filter efficiency, but also led to an increase in the packing density, which ultimately increased breathing resistance. Although most of the approaches for electrospun filters are based on single layers of nanofibers, the arrangement of the nanofiber layers have shown certain improvements in filtration and pressure drop [1,11,20,40]. Thus, in order to retain filtration efficiency and reduce breathing resistance, a combination of nanofiber layers, in alternate and symmetric stacking configurations, was studied. Thus, layers of PAN electrospun fibers were strategically disposed in a multilayer sandwich approach, as depicted in Figure 4. For the multilayer stacked structures, the use of PAN electrospun fibers with a basis weight of 0.4 and 0.8 g/m^2^ were used, as these resulted in high filtration materials as monolayers, and a pressure drop was deemed acceptable if extrapolated to 200 cm^2^ areas (i.e., area of a typical respirator). 

The results in filtration efficiency of the multilayer structures can be seen in Table 1. For mono and multilayer samples prepared with a total of 0.8 g/m^2^ of basis weight (distributed as 0.8 g/m^2^ for the monolayer and 0.4/0.4 g/m^2^ for the multilayer filters), a filtration efficiency over 99% was achieved, regardless of the layer distribution. For the distribution of 0.2/0.2 g/m^2^, the values were between 97–98%, with very similar values across the different stacked structures. 

**Figure 5:**
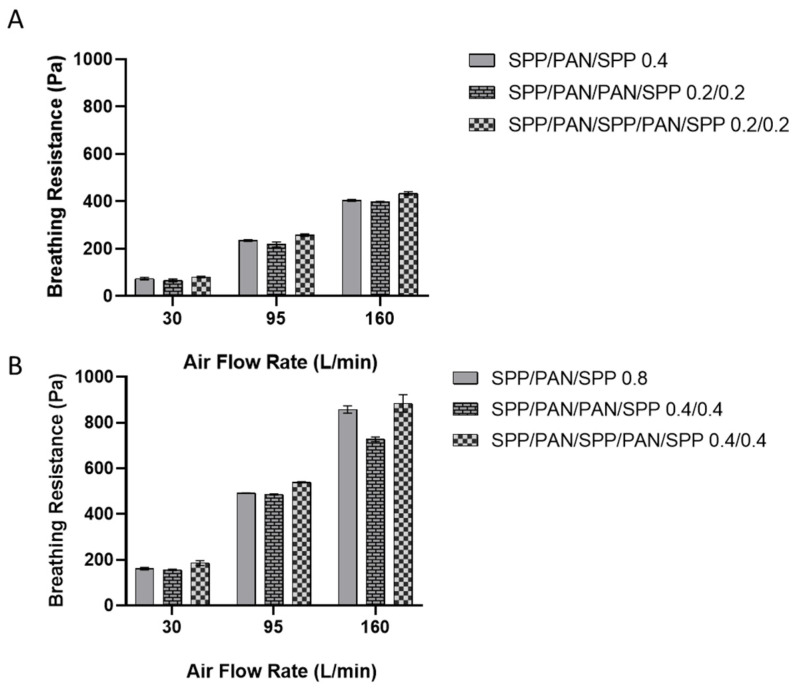
Breathing resistance at 30, 95 and 160 L/min of monolayer spunbond polypropylene/polyacrylonitrile/spunbond polypropylene (SPP/PAN/SPP), multilayer spunbond polypropylene/polyacrylonitrile/polyacrylonitrile/spunbond polypropylene (SPP/PAN/PAN/SPP) and spunbond polypropylene/polyacrylonitrile/spunbond polypropylene/polyacrylonitrile/spunbond polypropylene (SPP/PAN/SPP/PAN/SPP) filters with a total of 0.4 (**A**) and 0.8 (**B**) basis weight (g/m^2^), respectively. Results are presented as the mean ± SD (*n* = 3).

Figure 5A presents the breathing resistance at different flow rates for the monolayer and the multilayer structures made of a total of 0.4 g/m^2^ (distributed as 0.4 g/m^2^ for the monolayer and 0.2/0.2 g/m^2^ for the multilayer filters). The use of the symmetric multilayer structure (SPP/PAN/PAN/SPP) reduced slightly the breathing resistance at 30, 95 L/min and 160 L/min when compared to the monolayer structure. On the other hand, the alternate structure of SPP/PAN/SPP/PAN/SPP 0.2/0.2 g/m^2^ had an overall mild increase in breathing resistance for all the air flows when compared to the monolayer structure. For the case of the 0.8 g/m^2^ structures (Figure 5B), the improvement in breathing resistance at 160 L/min in the symmetric structure was much more accused than for the 0.4 g/m^2^ layers. Similar to what was previously observed, the alternating structure of SPP/PAN/SPP/PAN/SPP at the higher basis weight did not improve breathing resistance. Based on these results, it can be stated that filtration efficiency is maintained regardless of the disposition of the layers; however, a symmetric approach does result in an improvement in breathing resistance compared to their monolayer counterparts. 

Interestingly, by correlating the breathing resistance data with the percentage of porosity obtained in the mats (see Table 2), it can be seen that the percentage of porosity is reduced when a higher basis weight is deposited, which also translates to poorer breathability. Therefore, symmetric splitting of the fibers nanolayer is advantageous since it translates into an improvement in breathing resistance for the same amount of SPP used. This is attributed to the breaking up of the nanofibers layer, which leads to an overall increase of interfiber porosity.

### 3.3. Antimicrobial Activity of the Multilayer Electrospun Filters

The use of inorganic particles as a multifunction agent for nanofibrous mats has emerged over the past few years as a manner to create more effective materials in the areas of biomedicine, food and filtration [22,23,27,30,41]. In this work, to generate antimicrobial nanocomposite filter materials, loadings of 1, 3, 10, and 20 wt.% of ZnO nanoparticles were added to the PAN solution and were characterized in terms of viscosity and conductivity (see Table 3). 

**Figure 6:**
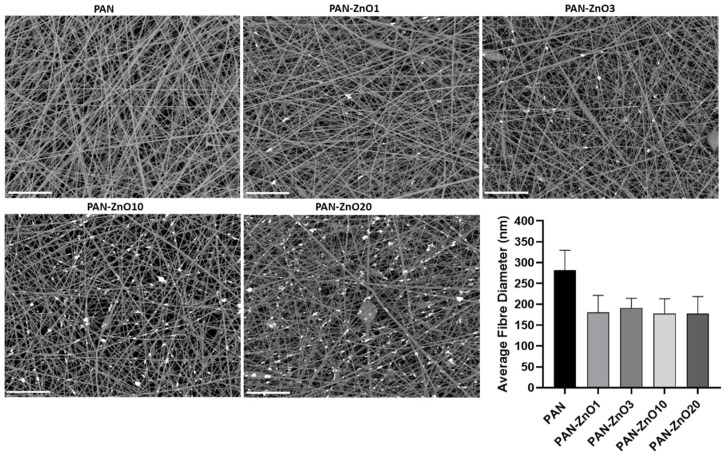
Field emission scanning electron microscope (FE-SEM) micrographs and average fiber diameter of the electrospun polyacrylonitrile (PAN) and PAN containing Zinc Oxide nanoparticles (PAN-ZnO) at 1 (PAN-ZnO1), 3 (PAN-ZnO_3_), 10 (PAN-ZnO10) and 20 (PAN-ZnO20) wt.%. Scale bar = 10 µm. Results are presented as mean ± SD (*n* = 3).

An increase in both viscosity and conductivity was observed with the increasing active filler loading. Interestingly, this increase in viscosity is not reflected in the fiber diameter. Thus, Figure 6 indicates that a reduction in fiber diameter was observed with the incorporation of the ZnO. PAN without the inorganic filler had a fiber diameter of 282.5 ± 47.6 nm, whereas the ZnO-PAN nanofibers had a diameter of approximately 200 nm for all concentrations. Although there are many parameters that can influence electrospinning processing and fiber morphology [42], it may be that the driving factor to control the nanofiber size in these experiments was the conductivity. The charge difference in the solution caused by the addition of ZnO could lead to a stretching effect of the solution jet, leading to a reduction in fiber diameter. This is in good agreement with previous reports based on the addition of ZnO to PAN solutions, in which the authors suggested that the reduction in the fiber diameter was due to the change in charge density of the PAN solution with the presence of ZnO [43]. From the FE-SEM micrographs, it can also be observed that the addition of ZnO particles led to the presence of isolated beads across the morphology of the fibers (see Figure 6). The presence of beaded fibers have shown benefits in air filtration, since beads can improve the permeability of the membrane and consequently lower the pressure drop [44]. 

The biocide capacity of the ZnO-loaded nanofibers was then analyzed. The antimicrobial effect of ZnO particles is exerted by different mechanisms of action such as the creation of reactive oxygen species (ROS) and ion release to the fiber surface (Zn^2+^), which could result in membrane dysfunction, interruption and blockage of transmembrane electron transport [45,46,47]. In some cases, the antimicrobial performance of ZnO has been reported to be triggered by humidity, which is advantageous considering that humidity will be present in the environment, and is also created by the respirator user during breathing. This feature also allows the particles to present a broad spectrum of inhibition for both Gram+ and G− bacteria [22].

In this regard, Table 4 gathers the *S. aureus* and *E. coli* reduction of the electrospun PAN filters containing ZnO particles at different concentrations (1, 3, 10 and 20 wt.%) after 24 h. The samples were tested with the nanofibers with the base weight of 0.4 g/m^2^. The filter material containing 1 wt.% of ZnO showed a strong reduction (R ≥ 3) against both types of bacteria, indicating a strong antimicrobial activity. In the case of the PAN-ZnO at 3 wt.%, the *S. aureus* and *E. coli* showed an even higher reduction. For the sample with 3 wt.% loading against *S. aureus*, no counts were measured, so by default a R > 3 was considered. On the other hand, the filters containing ZnO nanoparticles at 10 and 20 wt.% showed a strong antimicrobial activity (R ≥ 3), but there was an evident decrease in growth inhibition when compared with the filters containing ZnO at 3 wt.%. Such a decrease can be ascribed with nanoparticle agglomeration within the fibers due to excessive filler loading in the fibers. 

**Figure 7:**
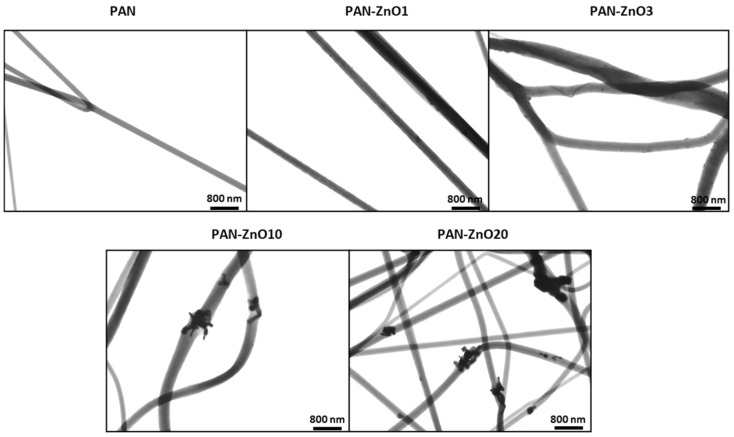
Transmission Electron Microscope (TEM) micrographs of polyacrylonitrile (PAN) and PAN containing Zinc Oxide (PAN-ZnO) nanoparticles at 1 (PAN-ZnO1), 3 (PAN-ZnO_3_), 10 (PAN-ZnO10) and 20 (PAN-ZnO20) wt.%.

Figure 7 shows the TEM micrographs of the ZnO-PAN loaded filters, confirming the presence of the ZnO nanoparticles efficiently encapsulated within the polymer fibers for all the tested ZnO contents. For the lower ZnO concentrations (PAN-ZnO1 and PAN-ZnO_3_), a relatively good dispersion and distribution across the fibers was observed. However, as the concentration increases, the presence of agglomerates becomes more pronounced, especially for PAN-ZnO20. These observations can be correlated with the reduction in antimicrobial activity reported in Table 4, since agglomerates are known to reduce the effective filler area, therefore diminishing their effectiveness as biocide agents [41]. Although some degree of agglomeration is common in nanocomposites due to limited solubility of dispersed nanoparticles, this phenomenon must be avoided as it can lead to reduced performance [27]. 

Similar findings have been reported by other authors, concluding that at high concentrations of nanoparticles, aggregates may form, decreasing the ZnO activity against bacteria [28,48,49]. For this reason, the generation of multilayer structures with ZnO particles were prepared at a concentration of 3 wt.%, as this concentration gave optimum antibacterial properties whilst being well dispersed and distributed across the fibers. Considering these results, filters of PAN-ZnO_3_ were selected and additionally measured at different time points of bacterial exposure (1, 3, 6, and 8 h), in order to determine the bactericidal kinetics of the nanofibers once microorganisms are stopped at the filter surface. Table 5 indicates that after just one hour, a significant reduction can be seen for both types of strains, followed by a R ≥ 3 after 3, 6, and 8 h, indicating a strong bactericide behavior, and, therefore, confirming the feasibility to use these materials as potential antibacterial filters in respirators. 

### 3.4. Filtration Performance of the Antimicrobial Multilayer Filters

**Figure 8:**
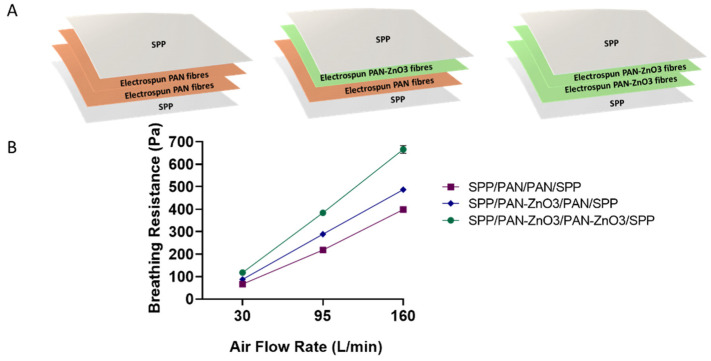
(**A**) Schematics of the multilayer filter configurations, spunbond polypropylene/polyacrylonitrile/polyacrylonitrile/spunbond polypropylene (SPP/PAN/PAN/SPP), spunbond polypropylene/polyacrylonitrile/polyacrylonitrile-zinc oxide/spunbond polypropylene (SPP/PAN-ZnO_3_/PAN/SPP) and spunbond polypropylene/polyacrylonitrile zinc oxide/polyacrylonitrile zinc oxide/spunbond polypropylene (SPP/PAN-ZnO_3_/PAN-ZnO_3_/SPP). (**B**) Breathing resistance of the above-mentioned configurations, SPP/PAN/PAN/SPP, SPP/PAN-ZnO_3_/PAN/SPP and SPP/PAN-ZnO_3_/PAN-ZnO_3_/SPP with a total of 0.4 basis weight (g/m^2^).

Once the antimicrobial performance of the filters was assessed, the breathing resistance and filtration efficiency were also analyzed. The biocide stacked filters were prepared as shown in Figure 8A. The stacked structures contained one or two layers of PAN-ZnO_3_ electrospun nanofibers with a total basis weight of 0.4 g/m^2^ (0.2/0.2 g/m^2^, see the schematics of the structures in Figure 8A). The filter efficiency was similar for the three conformations, with values of 97.01 ± 0.41% for the SPP/PAN/PAN/SPP configuration, 98.36 ± 0.01% for SPP/PAN-ZnO_3_/PAN/SPP and 99.38 ± 0.08% for PP/PAN-ZnO_3_/PAN-ZnO_3_/SPP configuration filter, suggesting that the presence of ZnO particles in either one or two layers of nanofibers slightly increased the filtration efficiency.

As per the breathing resistance (Figure 8B), the presence of one or two layers of PAN-ZnO_3_ fibers decreased the breathability proportionally; however, the values were still acceptable if extrapolated to the area of a conventional respirator (200 cm^2^), and especially for the configuration of SPP/PAN/PAN-ZnO_3_/SPP. Factors such as small and homogeneous pore diameters and high porosity are key factors to obtain a low breathing resistance material [23,50]. In this case, the presence of nanoparticles within the electrospun fibers led to a reduction in pore size associated with a lower fiber diameter, and thus an increase in pressure drop. This is consistent with the presence of one or two layers of PAN-ZnO_3_ fibers, obtaining a higher breathing resistance when two split nanofiber layers of PAN-ZnO were considered (Figure 8B).

Overall, these results indicate that ZnO-loaded PAN nanofibers are an effective technology to generate a highly efficient antimicrobial filter media for respirators, with values of up to 99% in filter efficiency, and relatively low breathing resistance (approximately 486.65–666 Pa at 160 L/min for 50 cm^2^ area, which would entail 120–170 Pa at 160 L/min in a 200 cm^2^ typical respirator area) and, moreover, with a strong antibacterial activity.

## 4. Conclusions

In this study, PAN electrospun nanofibers were deposited onto non-woven polypropylene as monolayer fibrous structures. A range from 0.1–1 g/m^2^ basis weight of nanofibers was deposited, and FFP2-type filtration efficiencies were obtained when the grammage was above 0.4 g/m^2^. In order to find a balance between efficiency and breathability, different configurations based on splitting and stacking of the nanofibers were carried out, which led to improved breathing resistance, especially with the symmetric splitting up of the nanofiber layer. To provide the filter with an antimicrobial activity, ZnO particles were added into the PAN solution. Although viscosity and conductivity increased with the presence of ZnO, both filter media with and without ZnO were prepared using the same electrospinning parameters. FE-SEM and TEM images confirmed the presence of ZnO particles dispersed and distributed throughout the fibers, and antimicrobial testing indicated a strong bactericidal activity, especially for PAN-ZnO_3_, i.e., with 3 wt.% content of the antimicrobial filler. In terms of filtration performance, the presence of ZnO particles did not diminish filtration efficiency, and although it increased somewhat the breathing resistance, the values remained low in accordance with what would be acceptable for the European EN149 norm for FFP2-type personal protective equipment (PPE). This study demonstrates the feasibility to generate a high filtration efficiency filter media with antimicrobial properties. It also suggests that the symmetric splitting up of the nanofiber layer is the optimal method to constitute the most efficient filters with this technology.

## 5. Patents

The conceptualization of this work is based on the patent P202030319.

## Figures and Tables

**Table 1:** Filtration efficiency (%) of the mono and multilayer configurations. Results are presented as the mean ± SD (*n* = 3).

Sample	Basis Weight (g/m^2^)	Filtration Efficiency (%)
SPP/PAN/SPP	0.8	99.84 ± 0.02
SPP/PAN/PAN/SPP	0.4/0.4	99.86 ± 0.03
SPP/PAN/SPP/PAN/SPP	0.4/0.4	99.91 ± 0.04
SPP/PAN/SPP	0.4	97.95 ± 0.46
SPP/PAN/PAN/SPP	0.2/0.2	97.01 ± 0.42
SPP/PAN/SPP/PAN/SPP	0.2/0.2	98.31 ± 0.17

**Table 2:** Mat porosity of the electrospun polyacrylonitrile fibers (PAN) at 0.2, 0.4 and 0.8 g/m^2^.

Sample	Basis Weight (g/cm^2^)	Mat Porosity (%)
PAN monolayer	0.2	88.86 ± 1.06
PAN monolayer	0.4	87.90 ± 0.58
PAN monolayer	0.8	74.39 ± 2.65

**Table 3:** Physicochemical properties (viscosity and conductivity) of polyacrylonitrile (PAN) and PAN containing Zinc Oxide (PAN-ZnO) nanoparticles at 1 (PAN-ZnO1), 3 (PAN-ZnO_3_), 10 (PAN-ZnO10) and 20 (PAN-ZnO20) wt.%.

Sample	Viscosity (cP)	Conductivity (µS/cm)
PAN	804.2 ± 0.95	65.98 ± 0.40
PAN ZNO1	808.9 ± 0.88	66.66 ± 0.87
PAN ZnO_3_	812.0 ± 3.53	69.79 ± 0.22
PAN ZNO10	924.85 ± 2.04	72.15 ± 1.54
PAN ZNO20	954.1 ± 1.11	72.89 ± 0.08

**Table 4:** Antibacterial activity of PAN containing Zinc Oxide (PAN-ZnO) nanoparticles at 1, 3, 10 and 20 wt.% against *Staphylococcus aureus* CECT240 (ATCC 6538p) and *Escherichia coli* CECT434 (ATCC 25922) after 24 h exposure. R corresponds to the reduction value.

Microorganism	ZnO (wt.%)	Control (PAN) Log (CFU/mL)	PAN-ZnO Log (CFU/mL)	R
*S. aureus*	1	8.11 ± 0.23	4.85 ± 0.18	3.26
	3		No Counts	R > 3
	10		3.20 ± 0.20	4.91
	20		2.85 ± 0.14	5.26
*E. coli*	1	8.01 ± 0.17	4.99 ± 0.12	3.02
	3		1.25 ± 0.15	6.76
	10		3.85 ± 0.23	4.16
	20		3.56 ± 0.17	4.45

**Table 5:** Antibacterial activity of polyacrylonitrile (PAN) containing Zinc Oxide (PAN-ZnO) nanoparticles at 3 wt.% against *Staphylococcus aureus* CECT240 (ATCC 6538p) and *Escherichia coli* CECT434 (ATCC 25922) after 1, 3, 6, and 8 h exposure. R corresponds to the reduction value.

Microorganism	Time (h)	Control Log (CFU/mL)	PAN-ZnO_3_ Log (CFU/mL)	R
*S. aureus*	1	6.01 ± 0.11	3.76 ± 0.21	2.25
	3	6.86 ± 0.17	3.68 ± 0.15	3.18
	6	7.18 ± 0.19	3.10 ± 0.18	4.08
	8	7.86 ± 0.13	2.99 ± 0.17	4.87
*E. coli*	1	5.98 ± 0.09	3.96 ± 0.11	2.02
	3	6.36 ± 0.10	3.29 ± 0.13	3.07
	6	7.01 ± 0.14	3.32 ± 0.10	3.69
	8	7.88 ± 0.11	3.28 ± 0.15	4.60

## Data Availability

Not applicable.
